# Medical Education in Turkey in Time of COVID-19

**DOI:** 10.4274/balkanmedj.galenos.2020.2020.4.003

**Published:** 2020-06-01

**Authors:** Burcu Tokuç, Gamze Varol

**Affiliations:** 1 Department of Public Health, Tekirdağ Namık Kemal University School of Medicine, Tekirdağ, Turkey

We are living in rare and unprecedented times. As the coronavirus disease 2019 (COVID-19) pandemic interrupted many things in the world, it also disrupted medical education. Although the main priority of medical educators, who are also healthcare workers, is to prevent an epidemic and provide patient care, medical education also requires intense attention during this period.

For about 30 years, many medical schools decreased their basic science curriculum and integrated clinical and basic sciences in all classes. They switched to a competency-based learning model and diversified evaluation methods (1). In Turkey, in recent years, medical faculties have been trying to transform medical education into individualized, virtual education by reducing classic lectures, using more technology for laboratory lessons, promoting active, self-learning, and integrating education.

In most medical schools in Turkey, during the first three years of education, students were in physical environments only for laboratory practices or small group lessons, like problem-based learning (PBL) discussions, and they did not attend traditional lectures. Their physical presence is mandatory for applications in clinical settings during the last three years. Also, their last 12 months are individualized by their participation in clinical rotations and internships.

However, the WHO published pandemics among the top ten health threats at the beginning of 2020. The measures taken by China and Canada in the SARS outbreak in the early 2000s are known, but medical schools all around the world were unprepared for the pandemic. In the 2003 SARS outbreak, some medical schools in China canceled their formal education and delayed exams. Similarly, Canada interrupted clinical clerkships and electives for up to six weeks (2). Despite the difficulties posed by the outbreak, attempts have been made to try to overcome it with new initiatives. In a medical school in China, online PBL was implemented to complete the curriculum (2). Although Turkey had preliminary plans regarding health services after the SARS and H1N1 pandemics, it has not made any arrangements for medical education.

In Turkey, as in the whole world, COVID-19 has affected the educational process. Social distancing is still the most effective preventive strategy for COVID-19 until a specific drug or vaccine is developed. To comply with this measure, students were not supposed to meet in lecture halls, classrooms, or PBL rooms. Although some aspects of education in many faculties have been individualized for “anytime/anywhere”-asynchronous learning in recent years, students must gather for lessons such as laboratory sessions, bedside practices, and case/patient presentations and discussions.

As a precaution, at the beginning of the COVID-19 pandemic, the Higher Education Institution (YÖK) envisaged the transition to distance education in medical faculties, as in all faculties (3). All medical faculties transitioned the theoretical lessons of the curriculum to an online format. Practice courses and exams were postponed being held in the summer term, and a new academic calendar was prepared for the summer term. Internship training, which is completely based on clinical practices, was delayed starting after the pandemic. These changes also required a strong technological infrastructure and technical staff for medical faculties.

Some questions come to mind immediately after these changes. These include: What exactly is the student’s role in the clinic?, Can the student contribute to the service in such a crisis?, and Is this type of education sufficient for professionalism? During natural disasters, such as the Marmara earthquake that Turkey lived through before, students continued their education and contributed to health services. Nevertheless, in a highly contagious pandemic like this, students may be potential vectors for COVID-19 and may transmit it to themselves (4). Other factors that limit students' education in the clinic are the cancelation of routine appointments and surgical procedures, lack of testing, and the shortage of personal protective equipment (4).

It is not clear how long this situation will last. Soon, there may be occasions when quarantines may be required, and social distance may always be necessary. Moreover, the main problem here is how to provide clinical training, bedside training, and other necessary training, which are essential components of medical education under these conditions.

As explained by the Ministry of Health, in Turkey, the COVID-19 pandemic will not cause a healthcare worker shortage (5). While many faculties do not want to shorten the internship period and early graduation, some faculties implement YÖK’s decision not to delay the graduation of the student (6).

Nevertheless, as Rose said, “The profound effects of COVID-19 may forever change how future physicians are educated” (4). In the last 50 years, the reduction of infectious diseases against chronic diseases, the ease of treatment of infectious diseases with the use of antibiotics has also directed medical education and the curriculum of medical schools to the diagnosis and treatment of chronic diseases in disease research, and both drug and vaccine development studies.

Moreover, as Aslan and Sayek emphasize in their article “Today’s medical education approach cannot really respond to the pandemic(s)”(7).

At all hazards, the COVID-19 pandemic reminded the world, especially medical educators, the importance of the social dimension of medicine. It has demonstrated the necessity of medical education that prioritizes basic health services with a holistic approach and trains physicians to a comprehensive health system (Universal Health Coverage). This pandemic process will provide an opportunity to review the curricula of medical schools. It will lead to issues such as public health, regional health management, and both infectious disease and epidemic management being brought to the agenda again. This renewed interest might prompt questioning of its density in the curriculum. Consequently, community oriented and community based medical education and social accountability of medical schools should be implemented to all stages of medical education (7).

As a continuing feature in the medical environment for generations, the view that physicians serve even when they are ill and prioritize the patient is seen as professional behavior. However, the situation has changed with COVID-19. Doctors working while they are ill, those who are asymptomatic or incubating the virus, can easily transmit the disease to their patients. Therefore, professionalism and altruism should be redefined in physician competencies, and potential effects of all activities should be considered, even if done generously and prioritization of the patient over the physician should be ended.

Crises present some solutions, along with problems. Even within the COVID-19 crisis, the medical education community must learn from its experience and produce practical solutions. While doing these, they think ahead and prioritize the scientific approach. Although updating the course materials and making them suitable for distance education and virtual learning is seen as a benefit of the transition, the results of the training in this way require a very careful evaluation afterward.

As with all things, medical education is an area open to change and development, and these changes accelerate under challenging times. Educators should analyze the effects of existing changes with students to identify the new educational principles and practices.
